# Improvement responses in patellar tendinopathy patients treated with ultrasound‐guided galvanic electrolysis technique: A 3‐year follow‐up study

**DOI:** 10.1002/jeo2.70323

**Published:** 2025-07-13

**Authors:** Ferran Abat, Carlos De la Fuente, Matias Roby, Alberto Schek, Santiago Roby, Jocelio Campos, Roberto Yañez

**Affiliations:** ^1^ Sports Orthopaedic Department ReSport Clinic, Pompeu Fabra University of Health Sciences Tecnocampus. GRACIS Research Group (GRC 01604) Barcelona Spain; ^2^ Exercise and Rehabilitation Sciences Institute, Postgraduate, Faculty of Rehabilitation Sciences Universidad Andres Bello, Universidad Andres Bello Santiago de Chile Chile; ^3^ Knee Orthopedics Service, Clinica MEDS Santiago Chile; ^4^ Innovation Center, Clinica MEDS Santiago Chile; ^5^ Sports Medicine Department Paracelsus Sportmedizin & Prävention Bremen Bremen Germany; ^6^ Sports Medicine Department ReSport Clinic Barcelona Spain

**Keywords:** anterior knee pain, electrotherapy, pain, patellar tendon, quadriceps, USGET

## Abstract

**Purpose:**

To determine the pattern changes over time in patients who experience different effects on the Victorian Institute of Sport Assessment–Patella score (VISA‐P), International Knee Documentation Committee (IKDC), Kujala score and Tegner activity score following 3 years of follow‐up after an ultrasound‐guided galvanic electrolysis technique (USGET) intervention in patellar tendinopathy.

**Methods:**

Fifty‐one adult patients with patellar tendinopathy were followed for 3 years after undergoing USGET. Clinical evaluations were conducted at baseline, 3‐, 6‐, 12‐, 24‐ and 36‐month postintervention, evaluating the VISA‐P, IKDC, Kujala score and Tegner activity score. The intervention was applied bimonthly for 3 months, accompanied by a biweekly standardised therapeutic exercises routine. Clinical outcomes were analysed with latent growth linear modelling (*α* = 5%), which assesses individual trajectories of change over time, capturing both initial status (intercepts) and rate of change (slopes) while accounting for variability across subjects.

**Results:**

The mean intercepts were 58.9 pts (95% confidence interval [CI]: 55.2–67.8 pts, *p* < 0.001), 60.0 pts (95% CI: 56.7–64.0 pts, *p* < 0.001), 60.9 pts (95% CI: 55.8–65.4 pts, *p* < 0.001) and 3.6 pts (95% CI: 2.9–5.3 pts, *p* < 0.001) for VISA‐P, IKDC, Kujala and Tegner activity score, respectively. The mean slopes were 6.3 pts (95% CI: 3.9–7.4 pts, *p* < 0.001), 5.7 pts (95% CI: 4.6–6.9 pts, *p* < 0.001), 5.1 pts (95% CI: 4.1–6.6 pts, *p* < 0.001) and 0.6 pts (95% CI: 0.4–1.0 pts, *p* < 0.001) for the VISA‐P, IKDC, Kujala and Tegner activity score, respectively.

**Conclusion:**

USGET treatment demonstrates functionality restoration and pain decrease over time. The inflection point of clinical improvement was at 6 months, followed by a steady phase. There were positive patterns of increase in the linear slopes for VISA‐P, IKDC, Kujala score and Tegner activity score observed in latent growth linear modelling.

**Level of Evidence:**

Level IV, case series with no comparison group.

**Abbreviations**
CI: confidence interval
IKDC: International Knee Documentation Committee score
IL‐1β: interleukin‐1 beta
PPAR‐γ: peroxisome proliferator‐activated receptor gamma
TNF‐α: tumour necrosis factor‐alpha
USGET: ultrasound‐guided galvanic electrolysis technique
VEGF: vascular endothelial growth factor
VISA‐P: The Victorian Institute of Sport Assessment‐Patella score

## BACKGROUND

Patellar tendinopathy accounts for approximately 10% of clinical knee diagnoses and often affects the quadriceps tendon, patellar tendon or both in active populations [28, 30]. It is also characterised by tendon pain [22] and degenerative changes, including tissue fragmentation, collagen alteration and vascular hyperplasia when tendinopathy is established [4, 23]. The ultrasound‐guided galvanic electrolysis technique (USGET) has shown promising clinical outcomes in both medium‐ [5] and long‐term follow‐ups [4, 27] in patellar tendinopathy patients. However, whether patients experience time course pattern improvements following USGET for patellar tendinopathy remains debated.

The USGET is an invasive ultrasound‐guided technique that induces a biochemical reaction through a nonthermal [6, 7] galvanic current (direct current with low voltage and amperage) in the damaged tissue [1]. In rat models, USGET has shown to decrease pro‐inflammatory mediators like tumour necrosis factor‐alpha and interleukin‐1 beta (IL‐1β), and increase the expression of anti‐inflammatory proteins and vascular endothelial growth factors like peroxisome proliferator‐activated receptor gamma (PPAR‐γ) and vascular endothelial growth factor (VEGF), respectively [5, 6]. These changes allow a controlled local inflammatory reaction followed by phagocytosis and regeneration promotion in the affected tissue [2, 6]. This local inflammation, phagocytosis and regeneration may support the effective outcomes observed in different tendinopathies reports [4, 7, 16, 17, 29, 31, 32].

A previous long‐term follow‐up of ten years [4] has categorised patellar tendinitis patients based on the Victorian Institute of Sport Assessment‐Patella score (VISA‐P), a questionnaire to assess the severity of patellar tendinopathies [18, 27]. One hundred points represent an asymptomatic athlete who can fully engage in sports, while the minimal score of 0 points represents the most affected patellar condition [18, 19]. The long‐term follow‐up of USGET in patellar tendinopathy patients categorised patients' responses for visual purposes, dividing at baseline patients with VISA‐P scores higher or lower than 50 points, resulting in heterogeneous standard deviation and means between the groups across the ten years of follow‐up [3]. Therefore, no clear time patterns of improvements and initial state of clinical patient outcomes such as VISA‐P, International Knee Documentation Committee score (IKDC), Kujala score, or Tegner activity score following a USGET intervention for patellar tendinopathy. These data are relevant to dose‐effectiveness studies and the timing of intervention effectiveness.

To better explore outcomes over time, more advanced repeated measurement techniques, such as latent growth curves, can estimate interindividual variability in intraindividual patterns of change [13]. Some advantages over traditional repeated measurements include the capacity to handle missing data, unequally spaced time points, nonnormally distributed or discretely scaled repeated measures, complex nonlinear or compound‐shaped trajectories, time‐varying covariates, multivariate growth processes, or higher levels of statistical power [13].

Consequently, we aimed to determine the patterns that change over time in patients who experience different effects on VISA‐P, IKDC, Kujala score and Tegner activity score following three years of follow‐up after a USGET intervention for patellar tendinopathy.

## METHODS

### Study design

In this prospective cohort study, patients with patellar tendinopathy were followed for 3 years after 3 months of intervention. They were clinically evaluated using the VISA‐P, IKDC, Kujala score and Tegner activity scores. Measurements were performed at seven time points (baseline, 3‐, 6‐, 12‐, 24‐ and 36‐month postintervention).

### Participants

**Figure 1:**
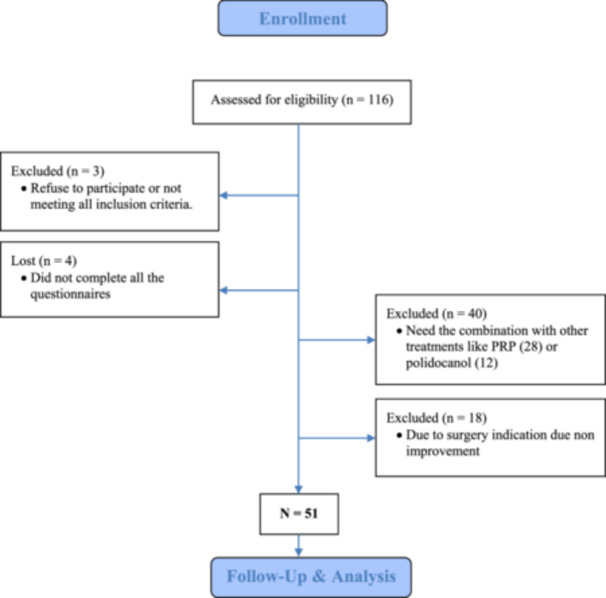
Enrolment, follow‐up and analysis of patients.

From August 2019 to April 2023, 51 adult patients with patellar tendinopathy (Table 1) were included in the study (Figure 1) based on standardised eligibility criteria previously described for patellar pathology follow‐up [3, 4]. These inclusion criteria were: (i) patients aged 18–60 years, (ii) a history of patellar tendon pain lasting for more than 4 weeks, (iii) patellar tendon tenderness to palpation, (iv) functional limitation due to patellar tendon pain, (v) musculoskeletal sonographic evidence of patellar tendon degeneration, (vi) injury at the inferior pole of the patella and (vii) Blazina classification ≥ grade I [3, 4]. The exclusion criteria were (i) chronic articular diseases > grade II Kellgren Lawrence, (ii) concomitant knee pathologies such as cruciate ligament injuries or meniscal tears, (iii) contraindications for USGET (pregnancy, prosthesis, osteosynthesis, cardiac disease, malign tumour, infection or coagulopathy) and (iv) concomitant administration of drugs like fluoroquinolones, anticoagulants, corticosteroids, or nonsteroidal anti‐inflammatory drugs [3, 4].

**Table 1:** Patient demographics.

Demographics	Total
Age (years)^a^	35.9 (4.5)
Sex (male: female)^b^	39:12
Weight (kg)^a^	72.5 (12)
Height (m)^a^	176.4 (6.8)
Body mass index (kg/m^2^)^a^	22.6 (2.6)
Physical activity (days/week)^a^	4.3 (1.0)
Physical activity (hours/day)^a^	1.8 (1.2)
Laterality (right:left)^b^	35:16
Symptoms duration (months)^a^	17.6 (8.2)

^a^ Data are expressed as mean (standard deviation).

^b^ Absolute frequencies.

The study was approved by the university institutional review board (*CER n.201912*) and conducted according to the Helsinki Declaration. All the patients signed written informed consent to participate in the study.

### Patellar physical examination and sonographic assessment

The same medical doctor (F.A.) made the clinical diagnosis with the patient lying supine; the knee was fully extended, and the quadriceps muscle was relaxed. Patients reported a history of insidious and recurrent pain onset when starting sports activities. During the physical exam, the evaluator identified pain at the insertion point on the lower pole of the patella through palpation. Additionally, a sudden and rapid contraction of the quadriceps muscle elicited pain.

**Figure 2:**
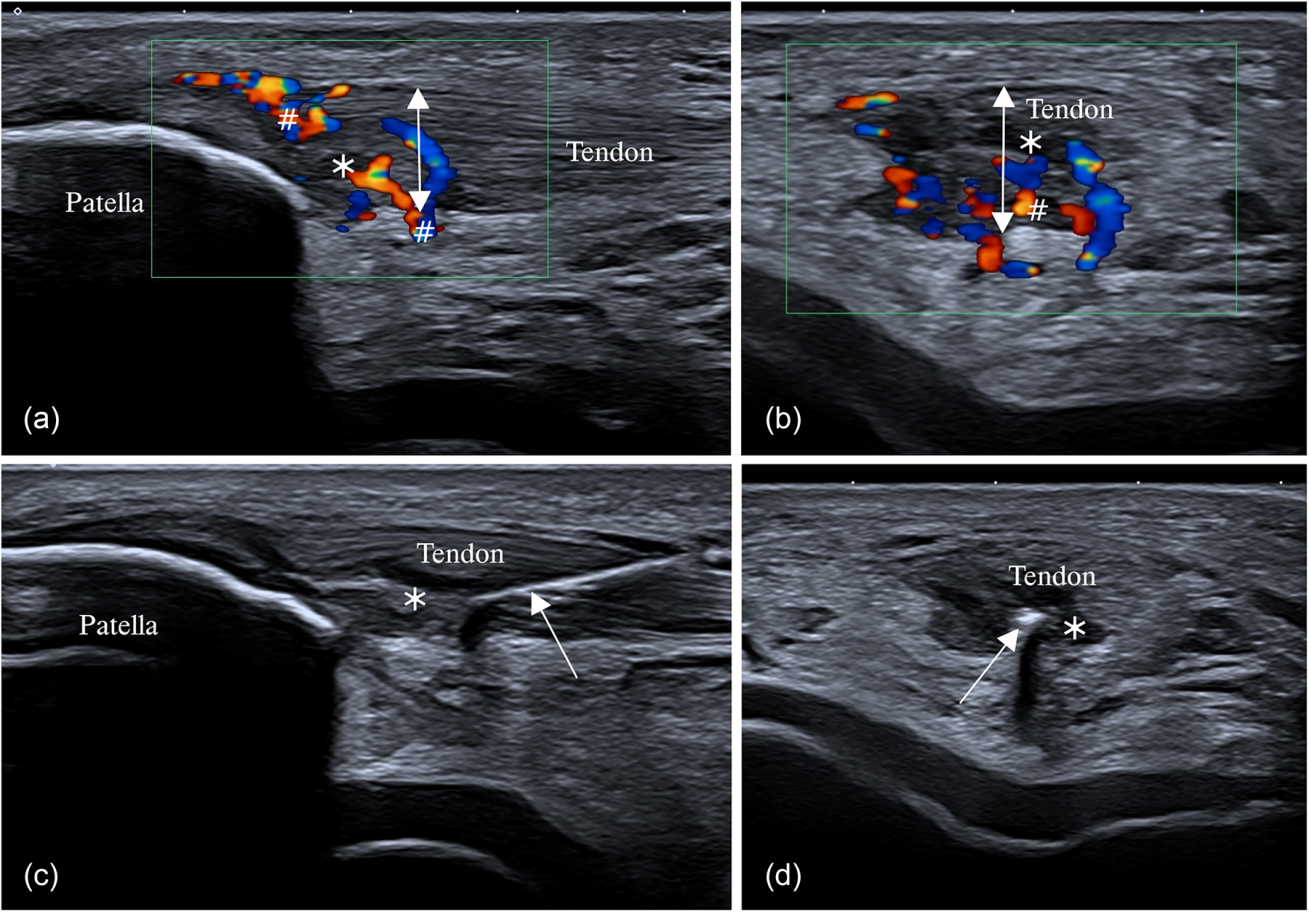
Image of high‐definition colour Doppler ultrasound with 6–15 MHz linear probe showing patellar tendinopathy. Longitudinal (a) and transversal view (b) of an injured patellar tendon were shown. The proximal patellar tendon has thickening (double‐headed arrow), showing structural damage and hypo‐echoic regions (^⁎^). The colour‐doppler analysis shows intensive hypervascularization inside and outside the dorsal side of the tendon (arrow). Images (c) and (d) show treatment using USGET (arrow) with ultrasound guidance along the longitudinal and transverse view, right at the lesion focus (^⁎^).

A high‐definition musculoskeletal ultrasound (US) machine of 14 MHz with a linear probe of 58 mm and Doppler colour was used to analyse hypervascular ingrowth within the injured tendon, allowing visualisation of blood flow [21]. Ultrasound demonstrated a sensitivity of 58% and a specificity of 94% for diagnosing patellar tendinopathy, performing comparably to MRI and very accurately treating the target area [34]. The ultrasound examination revealed hypoechogenicity and a disrupted fibrillar pattern in the patellar tendon, indicating disorganised connective tissue and tendon thickening (Figure 2). In certain cases, well‐defined hypoechoic images were observed, signifying intrasubstance ruptures with nodular or fibrillar shapes, typically appearing in the deeper region of the tendon near the lower pole adjacent to the patella. The middle segment of the tendon often exhibited thickening (greater than 4 mm) associated with hypoechoic areas, zones of disorganised collagen and loss of the fibrillar pattern. Cortical irregularities at the proximal patellar enthesis were also common. In some cases, paratenon involvement [14], adhesions to Hoffa's fat pad, and calcifications appeared as white hyperechogenic zones.

### USGET intervention

A USGET session was conducted bimonthly. During each session, the patient was positioned in a supine position with the knee at 20° flexion. The treatment area was disinfected with isopropanol before and after each session. Local anaesthesia was applied under aseptic measures (3 mL of Mepivacaine hydrochloride on the surface of the patellar tendon) to avoid pain during the USGET intervention. A sterile 0.3 mm stainless steel acupuncture needle (Agupunt) was used to administer the galvanic current. The electrolysis was carried out using a certified biomedical device (European Medical Device Regulation, MDR 2017/745, notified body CE0344) through the Acure 8000 device (GymnaUniphy NV). This biomedical device allows modulated galvanic electricity via the negative electrode cathodic flow. The electrolysis procedure was performed guided by ultrasound. The mean time dose was delivered for 120.7 s (with a minimum of 92.0 s and a maximum of 237.0 s). The current was mainly delivered in the inferior pole of the patella with an average current of 5.8 mA (with a minimum of 5 mA and a maximum of 8 mA). An average of 687.8 mC was applied (with a minimal dosage of 520 mC and a maximum of 860 mC) until the treated area was fully debrided.

The USGET intervention was accompanied by biweekly sessions of standardised therapeutic exercises [2, 3, 4]. Patients underwent ankle mobility, vibratory stimulation (focused on global exercises like Squat, Balance or single‐leg tasks), trunk stabilisation, hip reinforcement, quadriceps and foot and ankle muscle strengthening, balance training, plyometrics, agility mobility (from closed tasks to semiopen environments) and muscle strengthening prioritising using inertial (rotational) systems always prioritising unilateral work and adjusting the load according to the degree of tolerance.

### Functional patient outcomes

This study considered qualitative variables of functional patient outcomes (VISA‐P, IKDC, Kujala score and Tegner activity score) as standard measurements in patellar tendinopathy patients [2, 3, 5]. The VISA‐P score is a qualitative score with a resolution of 1 point, ranging from 100 points in asymptomatic patients to a minimum of 0 points, indicating the more compromised patellar tendons [19, 33]. The IKDC is a continuous numerical score ranging from 100 points in asymptomatic patients to a minimum of 0 points in the highest level of symptoms for each item [9, 10, 11]. The Kujala score is a qualitative score with a resolution of 1 point, ranging from 100 points in symptomatic anterior knee pain and with a minimum of 0 points, indicating asymptomatic patients [9, 10, 11]. The Tegner activity score is a qualitative self‐administered patient report measuring the work‐ and sports‐based activity level [10]. It has a resolution of 1 point and ranges from 0 to 10, where 10 points engage in highly competitive sports like rugby, football and soccer, while 0 points indicate a more compromised activity level. All data were collected by physical interview during the treatment period and through a telephone interview after the sixth month, as was previously reported [5, 6].

### Data analysis

The time course of functional patient outcomes (VISA‐P, IKDC, Kujala score and Tegner activity score) during 3 years of follow‐up are described as repeated measurement raincloud plots (median and interquartile range, distribution and connected samples) and summarised in Table 2 as median and interquartile range. A latent growth linear model was performed to analyse the effects of the time course trajectories at baseline and 1‐, 3‐, 6‐, 12‐, 24‐ and 36‐month follow‐up [12, 13]. The fit model was described through the chi‐square test (*χ* ^2^). The mean and variance factors for intercept and slope change were determined with their 95% confidence interval (95% CI) and *p*‐values. The effect size (*f* ^2^) was calculated as the ratio of the slope variance to the total variance (σ^2^_slope_/(σ^2^_slope_ + σ^2^_intercept_)) as an indicator of effect strength. Then, the *f* ^2^ was transformed to Cohen's *d* effect size using the transformation *d* = 2*(*f* ^2^)^0.5^, representing the standardised mean difference between the basal measure and time‐dependent changes.

Latent covariances were determined with their estimates, 95% CI, and *p*‐values. The coefficient of determination (*R* ^2^) was described for each time point. Finally, the fit indexes of comparative fit Index (CFI), Tucker–Lewis index (TLI), Bentler–Bonett nonnormed fit index (NNFI), Bentler–Bonett normed fit index (NFI), parsimony normed fit index (PNFI) and Bollen's incremental fit index (IFI) were determined. All tests used an *α* equal to 5%, and all estimations were performed through the open‐source software JASP 0.18.3 (University of Amsterdam, Netherlands).

## RESULTS

**Figure 3:**
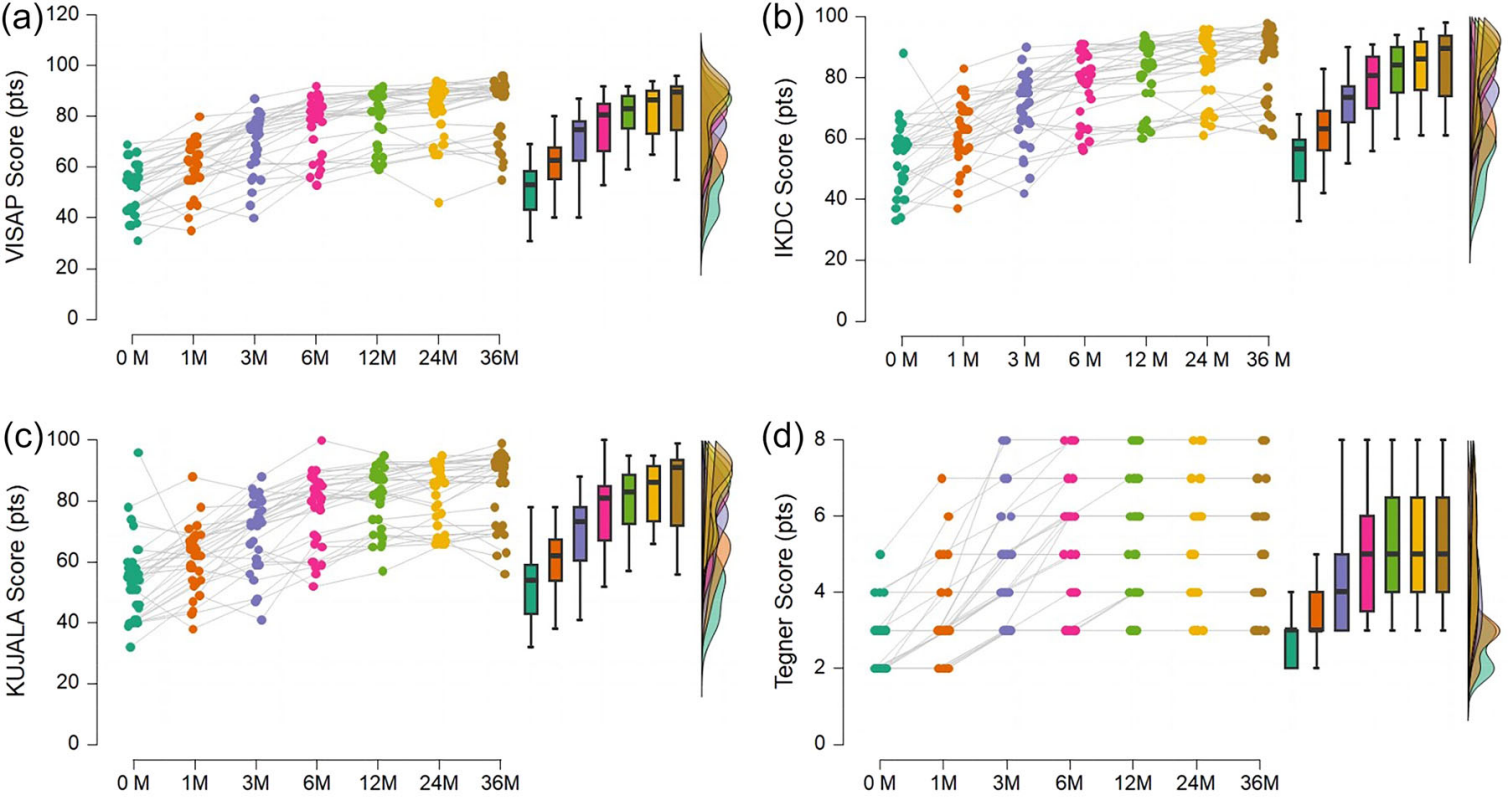
Time series of the Victorian Institute of Sport Assessment‐Patella score (VISA‐P) (a), International Knee Documentation Committee score (IKDC) (b), Kujala score (c) and Tegner activity score (d) from 0 and 36 months. Each time point is represented by different colours. Each raincloud plot combines a scatter plot (each sample), box plot (descriptive statistics), and density plot (distributions), providing a comprehensive visualisation of the time‐series patterns.

The time series for VISA‐P, IKDC, Kujala score and Tegner activity score are described in Figure 3. There was statistical significance for the growth curve model fit for VISA‐P (*χ* ^2^ = 218.1, *p* < 0.001), IKDC (*χ* ^2^ = 162.4, *p* < 0.001), Kujala score (*χ* ^2^ = 143.1, *p* < 0.001), Tegner activity score (*χ* ^2^ = 875.9, *p* < 0.001). The latent growth curve outcomes are described in Table 2.

**Table 2:** Latent growth curve outcomes.

Latent curve	Intercept	*p* value	Linear slope	*p* value	ES
VISA‐P					
Mean	58.9 (95% CI: 55.2–67.8)	<0.001	6.3 (95% CI: 3.9–7.4)	<0.001	0.58
Variance	59.5 (95% CI: 28.3–103.4)	<0.001	3.1 (95% CI: 0.2–6.5)	0.013	0.44
IKDC					
Mean	60.0 (95% CI: 56.7–64.0)	<0.001	5.7 (95% CI: 4.6–6.9)	<0.001	0.59
Variance	57.9 (95% CI: 25.8–89.9)	<0.001	4.2 (95% CI: 1.6–7.2)	0.005	0.53
Kujala score					
Mean	60.9 (95% CI: 55.8–65.4)	<0.001	5.1 (95% CI: 4.1–6.6)	<0.001	0.57
Variance	31.0 (95% CI: 26.7–93.2)	<0.001	1.7 (95% CI: −0.2 to 3.8)	0.057	0.34
Tegner score					
Mean	3.6 (95% CI: 2.9–5.3)	<0.001	0.6 (95% CI: 0.4–1.0)	<0.001	0.76
Variance	34.9 (95% CI: 40.4–72.9)	<0.001	1.5 (95% CI: 0.1–4.7)	<0.001	0.40

Latent cov.	Estimate	Std. error	95% CI	*p* value
VISA‐P	2.14	3.47	−6.5 to 7.3	0.538
IKDC	1.96	3.36	−4.3 to 7.9	0.560
Kujala score	7.47	3.51	3.5–19.4	0.033
Tegner	6.93	1.37	0.0–22.2	<0.001

*R* ^2^	Basal	1 month	3 months	6 months	12 months	24 months	36 months
VISA‐P	0.336	0.681	0.705	0.860	0.975	0.850	0.722
IKDC	0.287	0.685	0.694	0.816	0.926	0.951	0.857
Kujala score	0.375	0.774	0.852	0.883	0.947	0.940	0.893
Tegner	0.323	0.985	0.941	0.960	0.993	0.979	0.987

Fit indices	CFI	LTI	NNFI	PNFI	IFI	RNI
VISA‐P	0.541	0.581	0.581	0.560	0.539	0.541
IKDC	0.616	0.649	0.649	0.632	0.614	0.616
Kujala score	0.764	0.784	0.784	0.799	0.763	0.764
Tegner	0.576	0.613	0.613	0.623	0.576	0.576

Abbreviations: CFI, comparative fit index; Cov., covariances; E.S., Cohen's d effect size; IFI, Bollen's incremental fit index; IKDC, International Knee Documentation Committee; NFI, Bentler–Bonett normed fit index; NNFI, Bentler–Bonett nonnormed fit index; PNFI, Parsimony normed fit index; *R* ^2^, coefficient of determination; RNI, relative noncentrally index; TLI, Tucker–Lewis index; VISA‐P The Victorian Institute of Sport Assessment‐Patella.

The VISA‐P showed a significant mean initial state different from zero (58.9 pts [95% CI: 55.2–67.8 pts], *p* < 0.001) with significant within‐subject variance (59.5 pts [95% CI: 28.3–103.4 pts], *p* < 0.001). The VISA‐P showed a significant and the highest mean linear growth (improvement) over the follow‐up (6.3 pts [95% CI: 3.9–7.4 pts], *p* < 0.001) with significant within‐subject variance (3.1 pts [95% CI: 0.2–6.5 pts], *p* < 0.013).

The IKDC showed a significant mean initial state different from zero (60.0 pts [95% CI: 56.7–64.0 pts], *p* < 0.001) with significant within‐subject variance (57.9 pts [95% CI: 25.8–89.9 pts], *p* < 0.001). The IKDC showed a significant mean linear growth (improvement) over the follow‐up (5.7 pts [95% CI: 4.6–6.9 pts], *p* < 0.001) with significant within‐subject variance (4.2 pts [95% CI: 1.6–7.2 pts], *p* = 0.005).

The Kujala score showed a significant mean initial state different from zero (60.9 pts [95% CI: 55.8–65.4 pts], *p* < 0.001) with significant within‐subject variance (31.0 pts [95% CI: 26.7–93.2 pts], *p* < 0.001). The Kujala score showed a significant mean linear growth (improvement) over the follow‐up (5.1 pts [95% CI: 4.1–6.6 pts], *p* < 0.001) without significant within‐subject variance (1.7 pts [95% CI: −0.2 to 3.8 pts], *p* = 0.057).

The Tegner score showed a significant mean initial state different from zero (3.6 pts [95% CI: 55.8–65.4 pts], *p* < 0.001) with significant within‐subject variance (34.9 pts [95% CI: 40.4–72.9 pts], *p* < 0.001). The Tegner score showed a significant mean linear growth (improvement) over the follow‐up (0.6 pts [95% CI: 0.4–1.0 pts], *p* < 0.001) with significant within‐subject variance (1.5 pts [95% CI: 0.1–4.7 pts], *p* < 0.001).

## DISCUSSION

The main findings of this study indicate that patients who underwent USGET intervention showed (i) improvement in clinical outcomes over time across all measured clinical scores, including VISA‐P, IKDC, Kujala score and Tegner activity score. (ii) The VISA‐P score exhibits the most significant changes over time, suggesting it is a more sensitive tool for measuring the USGET clinical effects on patellar tendinopathy compared to the IKDC, Kujala score and Tegner activity score. (iii) A steady phase of clinical improvement was observed beginning after the following 6 months, which was sustained over time until the 3rd year. (iv) Based on the latent covariate's outcomes, a higher baseline Tegner score suggests an association with a steeper improvement over time. Therefore, our findings suggest that USGET improves functional and pain scores item of scores on degenerated patellar tendons over time based on significant and positive growth slopes and strong fit indexes, where USGET treatment by 3 months is promising with long‐term clinical effects. Previous research has primarily focused on short‐term effects. For instance, de la Cruz Torres et al. [12] reported immediate autonomic responses in soccer players on the patellar tendon and USGET, while Borrella‐Andrés et al. [8] found no acute thermal effect in cadaveric models in the patellar tendon using USGET. Additionally, Abat et al. [4] compared USGET with conventional electrotherapy over a 60‐day period, demonstrating improvements but without long‐term follow‐up. Our study extends this body of knowledge by providing a three‐year longitudinal analysis, demonstrating clinical improvements in a patellar tendinopathy cohort.

The findings from the VISA‐P, IKDC, Kujala and Tegner activity scores provide insights into novel improvement patterns over time in patients undergoing USGET for patellar tendinopathy. These findings delineate a therapeutic window of 0–6 months, during which the most significant changes are observed where an inflection or elbow point is observed across each analyzed time series (median of 82 pts in VISA‐P, 82.5 pts in IKDC, 82 pts in Kujala and 5.5 pts in Tegner). Following this period, patients achieved higher scores, indicating a sustained period of high with partial functional recovery. Particularly, the Kujala score, which involves pain‐scoring measurements related to anterior knee pain [26] is improved, indicating a pain decrease after 3 years and changes between baseline and 6 months.

In this study, the VISA‐P questionnaire was shown to be the most sensitive tool for detecting the observed clinical improvements compared to the other scores based on their higher mean linear slope growth (6.3 in VISA‐P, 5.6 in IKDC, 5.1 in Kujala and 0.6 in Tegner). The median score increased from 53.5 pts at baseline to 89.5 pts at 3 years, which represents 2.8 times the minimal detectable change of 13 pts estimated in clinical settings for VISA‐P [20]. This change is indicative of patients starting from significant patellar dysfunction changing to a nearly asymptomatic tendon (>80 pts in VISA‐P [9]). This clinically significant improvement was observed as early as 6 months when patients obtained a median score of 82.0 points, crossing the functional threshold of 80 pts [9]. Previous literature has also shown a general trend in favour of using USGET, suggesting that USGET offers a more rapid recovery, consistent with our findings [4, 28]. Other therapeutical strategies, such as eccentric exercises, have shown comparable improvements [11], while interventions like platelet‐rich plasma have shown lower improvements than those observed in our study [24].

The steady phase of improvements followed for 6 months was sustained over time until the 3rd year. The median clinical scores ranged between 82.0 and 89.5 pts in VISA‐P, 82.5 and 89.0 pts in IKDC, 82 and 91 pts in Kujala, and 5.5 and 5.0 pts in Tegner, which demonstrate a low dispersion of the obtained high clinical scores between 6 and 36 months. These improvements refer to the significant restoration of knee functionality following the USGET treatment, in accordance with significant improvements in progressive loading interventions after 6 months [11] and minimally invasive treatments [28].

Based on the latent covariate's outcomes, a higher baseline Tegner score suggests an association with a steeper improvement over time. A higher Tegner score indicates patients are more independent in performing work and sports activities, which tend to obtain higher scores, while lower Tegner scores tend to obtain lower improvements during the follow‐up. Our findings show improvements of around 2–3 pts in the Tegner activity score in accordance with previous reports [9].

Our study is not beyond limitations. The main identified limitation was the lack of a control group, making it difficult to distinguish the specific effects of USGET from the natural course of tendon healing. However, tendons affected by tendinopathy typically remain in a state of chronic inflammation and pathological remodelling rather than undergoing spontaneous healing and returning to a healthy state. They do not heal like normal tendons after an acute injury. Therefore, the consistent improvements observed across different clinical scores following USGET are promising results, though limited. Readers should interpret our findings with caution and critical thinking. Another consideration is that the sample did not include patients requiring surgery, which likely have a worse prognosis and require a special further investigation. On the other hand, the identified therapeutical epoch between 0 and 6 months suggests being the main period where USGET induces tendon changes through biochemical reactions induced by galvanic current effects [1, 5, 6], allowing a controlled local inflammatory reaction, phagocytosis and regeneration promotion on the affected tissue [2, 4, 6, 7, 16, 17, 29, 31, 32]. Thus, this epoch would be an important period of therapeutic interest to optimise the long‐term results. However, future studies in pathological tendons using USGET should explore the dose effectiveness (there is a lack of consensus in the published literature) and the complementation with neurophysiological strategies to improve the remnant clinical impairments and restore the neuromuscular control [15] and pathological stress balance over pathological tendons [25].

## CONCLUSION

USGET treatment for patellar tendinosis restores functionality and decreases pain over time with an inflection point of improvement at 6 months, obtaining a steady phase of clinical improvement after 6 months. An increase in the linear slopes for VISA‐P, IKDC, Kujala score and Tegner activity score was observed in latent growth linear modelling. Our study showed outcomes surpassing or comparable to the best conservative treatments available in the literature.

## AUTHOR CONTRIBUTIONS

*Conceived and designed the experiments*: Ferran Abat. *Performed the experiments*: Ferran Abat, Jocelio Campos and Alberto Schek. *Analyzed the data*: Carlos De la Fuente, Roberto Yañez and Jocelio Campos. *Contributed analysis tools*: Carlos De la Fuente and Roberto Yañez. *Wrote the paper*: Carlos De la Fuente and Ferran Abat. *Manuscript review and correction*: Carlos De la Fuente, Ferran Abat, Alberto Schek and Roberto Yañez. All authors read and approved the final manuscript.

## CONFLICT OF INTEREST STATEMENT

Ferran Abat invented the USGET device (OEPM registration n.ES1.247.869U) and receives royalties from GymnaUniphy for scientific consulting and product development. This financial relationship has not influenced the design, conduct, interpretation, or reporting of the research presented in this manuscript. The remaining authors declare no conflicts of interest.

## ETHICS STATEMENT

This study was approved by the university institutional review board (CER n.201912). The datasets used and/or analyzed during the current study are available from the corresponding author on reasonable request. All the patients signed the written informed consent to participate in the study.

## Data Availability

Data are available on request due to privacy/ethical restrictions.
